# Computational study of the effects of arterial bifurcation on the temperature distribution during cryosurgery

**DOI:** 10.1186/s12938-018-0438-z

**Published:** 2018-01-16

**Authors:** Yong-Chang Zheng, Jun-Hong Wu, Zhi-Zhu He, Shao-Jiong Huang

**Affiliations:** 1 Department of Liver Surgery, Peking Union Medical College Hospital, Chinese Academy of Medical Sciences and Peking Union Medical College, Beijing, 100730 China; 2 College of Environmental and Energy Engineering, Beijing University of Civil Engineering and Architecture, Beijing, 100044 China; 3 Vehicle Engineering, College of Engineering, China Agricultural University, Beijing, 100083 China

**Keywords:** Bioheat transfer, Cryosurgery, Numerical simulation, Arterial bifurcation, Blood thermal effect

## Abstract

**Background:**

Thermally significant blood flows into locally cooled diseased tissues and warm them during cryosurgery so that the iceball is often hard to cover the whole diseased volume. This paper is aimed at investigating the effects of large arterial bifurcation on the temperature distribution during cryosurgery through simulation method.

**Methods:**

A parametric geometry model is introduced to construct a close-to-real arterial bifurcation. The three-dimensional transient conjugate heat transfer between bifurcated artery and solid tissues with phase change during cryosurgery is performed by finite volume method.

**Results:**

The discussion was then made on the effects of the relative position between cryoprobe and artery bifurcation, the inlet velocity of root artery and the layout of multiple cryoprobes on the temperature distribution and iceball evolution. The results show that the thermal interaction between blood flow and iceball growth near bifurcation is considerable complex. The thermal effects of bifurcation could modulate the iceball morphology, severely weaken its freezing volume and prevent the blood vessel from being frozen.

**Conclusion:**

The present work is expected to be valuable in optimizing cryosurgery scheme of the situation that the bifurcated artery is embedded into the disease tissue.

## Background

Cryosurgery has been widely demonstrated as an excellent therapeutic approach to destroy the diseased tissues (such as tumor) due to its minimal invasiveness [1–3]. In order to freeze and kill the target cells, largely decreasing the temperature of target tissues to below 0 °C is necessary. The temperature distribution in target tissue is the major parameter to evaluate the cryosurgical output. Accordingly, it is very important to accurately control the temperature distribution in order to enhance the destruction of diseased tissues and avoid the injury of healthy tissues.

Blood flow could remarkably affect the temperature distributions during freezing, especially with the presence of large blood vessels (larger than 0.5 mm in diameter) [4]. Thermally significant blood flows into locally cooled diseased tissues and warm them during cryosurgery so that the iceball is often hard to cover the whole diseased volume. Recently the study on thermal behavior of large blood vessels has attracted much attention in the cryosurgery area. Deng et al. [5] adopted infrared thermography system to investigate the thermal effects of large vessels during cryosurgery based on simulation and animal experiments. The results showed that the heating nature of the flowing blood in the large vessels could produce steep temperature gradients and inadequate cooling to the frozen tissues. Numerical simulation method was also used to study the thermal effects of the large blood vessel. In the early research [6], the blood velocity in the line-like vessel was simply considered as constant. The cylindrical cryoprobes and blood vessel were both approximated as cubes. Such simplification would induce numerical errors to the temperature distribution [7]. Latterly, a finite element method based on FEM commercial software was introduced to obtain a more accurate numerical solution of temperature field near blood vessel [8]. However, compared with the investigation for the thermal effects of large vessels on the hyperthermia ablation [9–14], study on the similar issues in cryosurgery is still rare.

Compared to single large line-like artery, the bifurcated artery (such as inlet artery in liver) has complicated structure so that the complex blood flow distribution would induce heterogeneous heat transfer surrounding artery bifurcation. In recent years, some investigations focus on the cooling effects of artery bifurcation on the hyperthermia ablation, such as microwave ablation [15, 16] and radio-frequency ablation [17]. However, few investigation contributes to the warm effects of artery bifurcation on the cryosurgery. The details of three-dimensional transient temperature distribution during cryosurgery surrounding artery bifurcation, which is more real and complex, are still unknown. The phase-change heat transfer combined with the convective mechanism of blood flow in arterial bifurcation during cryosurgery would be more complex than that during hyperthermia ablation. Tremendous contributions are needed to probe into such important issues, which are very useful for optimizing cryosurgery scheme of the situation that the bifurcated artery is embedded into the disease tissues. The aim of this paper is to disclose the detailed temperature characteristics of cryosurgery in the vicinity of an arterial bifurcation.

In present study, a parametric geometry model [18] is introduced to construct a close-to-real bifurcated artery. The steady state blood flow is considered here. The three-dimensional transient conjugate heat transfer between bifurcated artery and solid tissues during cryosurgery is performed by finite volume method. Then the relative position between cryoprobe and artery bifurcation, the changes of root vessel inlet velocity and the layout of multiple cryoprobes would be considered to investigate the detailed temperature distribution between bifurcation and cryoprobe. The mechanism of heat transfer between artery bifurcation and solid tissues, the iceball edge evolution would be then revealed to evaluate the thermal effects of large artery bifurcation on the tissue temperature distribution and iceball growth during cryosurgery.

## Geometric and mathematical model

### Geometric model

**Fig. 1:**
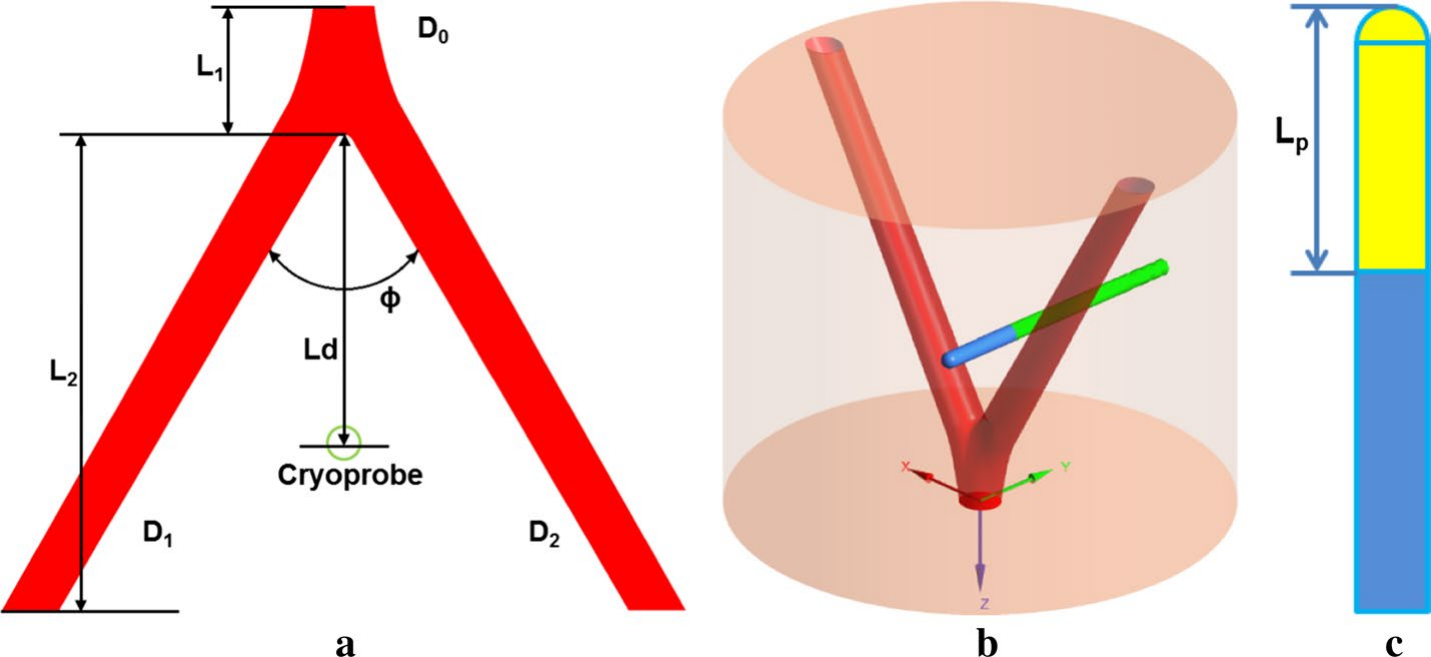
Schematic diagram of the bifurcated artery (**a**) and computational field (**b**). The shape of probe is illustrated in (**c**)

The arterial bifurcation is constructed by a parametric model [18], which is represented by two curved tubes with the same size attached to a straight root tube. It could reproduce effectively realistic configuration of arterial bifurcation. The thickness of artery wall is omitted here. The detailed geometry of symmetric arterial bifurcation is illustrated in Fig. 1a, where L_1_ = 20 mm is the distance from root vessel inlet to bifurcation, L_2_ = 80 mm is the height of daughter vessel along vertical direction, D_0_ = 10 mm is the diameter of root vessel [17], ${\text{D}}_{1} = {\text{D}}_{2} = \sqrt[3]{{{1 \mathord{\left/ {\vphantom {1 2}} \right. \kern-0pt} 2}}}{\text{D}}_{0}$ 7.94 mm is the diameter of daughter vessels, Ф = 60° is the angle of bifurcation. The arterial bifurcation is embedded in cylindrical solid tissues with diameter D_*t*_ = 120 mm and length L_*t*_ = L_1_ + L_2_ = 100 mm (see Fig. 1b). Single cylindrical cryoprobe with diameter D_p_ = 4 mm inserts the solid tissue near the bifurcation site along horizontal direction. The distance from cryoprobe center to bifurcation point denotes as L_d_ (see Fig. 1a). The probe shape is illustrated in Fig. 1c. The cryoprobe has two parts, adiabatic shaft and active tip with length L_p_ = 22 mm, which could lead to an extremely low temperature (such as − 196 °C of liquid nitrogen).

### Governing equation

During cryosurgery, the solid tissues consist of unfrozen region (*T* > *T*_*u*_), transition field (*T*_*l*_ ≤ *T* ≤ *T*_*u*_*)* and frozen area (*T* < *T*_*l*_*)* according to temperature distribution, where *T*_*l*_ and *T*_*u*_ denote respectively the lower and upper phase transition temperature of solid tissues. Then the Pennes bioheat transfer equation for whole solid tissues based on the effective heat capacity method [6] could be written as

1 $$\hat{C}\frac{{\partial T_{t} }}{\partial t}\, = \,\nabla \cdot \left( {\hat{\kappa }\nabla T_{t} } \right)\, + \,\hat{\omega }_{cb} C_{b} \left( {T_{cb} \, - \,T_{t} } \right)\, + \,\hat{Q}_{m} ,$$

*T*_*t*_ is the temperature of solid tissues and *T*_*cb*_ denotes the temperature of blood perfusion from capillary vessel. *C*_*b*_ is the heat capacity of blood. In addition, the detailed expression of $\hat{C}$ the effective tissue heat capacity, $\hat{\kappa }$ the effective tissue thermal conductivity, $\hat{\omega }_{cb}$ the effective blood perfusion and $\hat{Q}_{m}$ the effective tissue metabolic heat generation are determined by solid tissue state, which is given by

2 $$\hat{C}\, = \,\left\{ \begin{aligned} C_{f} \quad \quad T\, < \,T_{l} \hfill \\ \frac{{Q_{f} }}{{T_{u} \, - \,T_{l} }}\, + \,\left[ {C_{f} + \left( {C_{u} \, - \,C_{f} } \right)\frac{{T\, - \,T_{l} }}{{T_{u} \, - \,T_{l} }}} \right]\quad T_{l} \, \le \,T\, \le \,T_{u} \hfill \\ C_{t} \quad \quad T\, > \,T_{u} \hfill \\ \end{aligned} \right.,$$

3 $$\hat{\kappa }\, = \,\left\{ \begin{aligned} & \kappa_{f} \quad \quad T\, < \,T_{l} \\ & \kappa_{f} \, + \,\left( {\kappa_{u} \, - \,\kappa_{f} } \right)\frac{{T\, - \,T_{l} }}{{T_{u} \, - \,T_{l} }}\quad T_{l} \, \le \,T\, \le \,T_{u} \\ & \kappa_{t} \quad \quad T\, > \,T_{u} \\ \end{aligned} \right.,$$

and,

4 $$\hat{\omega }_{b} \, = \,\left\{ \begin{aligned} & 0\quad \quad T\, < \,T_{l} \hfill \\ & 0\quad \quad T_{l} \, \le \,T\, \le \,T_{u} \hfill \\ & \omega_{b} \quad \quad T\, > \,T_{u} \hfill \\ \end{aligned} \right.,\quad \hat{Q}_{m} \, = \,\left\{ \begin{aligned} & 0\quad \quad T\, < \,T_{l} \hfill \\ & 0\quad \quad T_{l} \, \le \,T\, \le \,T_{u} \hfill \\ & Q_{m} \quad \quad T\, > \,T_{u} \hfill \\ \end{aligned} \right.,$$

The thermo-physical parameters involved in the above equation could be found in [6]:*C*_*t*_ = *C*_*b*_ = 3.6 MJ/m^3^K, *C*_*f*_ = 1.8 MJ/m^3^K, *ω*_*cb*_ = 5 × 10^−4^/s, *Q*_*m*_ = 420 J/m^3^, *κ*_*f*_ = 2 W/mK, *κ*_*t*_ = *κ*_*b*_ = 0.5 W/mK, *L*_*f*_ = 250 MJ/m^3^, T_cb_ = 37 °C, *T*_*u*_ = − 1 °C and *T*_*l*_ = − 8 °C. Blood viscosity is *μ* = 2.5 × 10^−3^ Ns/m^2^.

The blood is modeled as incompressible and Newtonian flow, which is governed by Navier–Stokes equation. In the present study, the blood in bifurcated artery domain has large undercooling and could not be frozen due to flow. Then the blood velocity here is considered as steady and independent on the temperature. The energy equation for artery domain could be given as

5 $$C_{b} \left( {\frac{{\partial T_{b} }}{\partial t}\, + \,V \cdot \nabla T_{b} } \right)\, = \,\nabla \cdot \left( {\kappa_{b} \nabla T_{b} } \right)$$

where *T*_*b*_ denotes the temperature in bifurcated artery. The heat transfer in solid tissues described by Eq. (1) and bifurcated artery presented in Eq. (5) could be combined by the interface between them, which is a typical conjugate heat transfer problem.

### Boundary condition

For solid tissues boundary, the thermal condition is considered as adiabatic wall boundary. The inlet velocity of the root artery adopts parabolic velocity profile *u*(*r*) = 2 V[1 − (2*r*/*D*_0_)^2^], where V is average velocity and *r* the radial position. The inlet temperature of the root artery assumes as constant *T*_0_ = 37 °C. The outlet boundary of the daughter bifurcation artery adopts the pressure boundary, where the reference pressure is set as zero. The interface between solid tissues and cryoprobes tip is set as *T*_*p*_ = − 196 °C.

### Finite volume analysis tool

Firstly, the geometrical model of bifurcated artery constructed from software Solidworks imports into mesh generation software Gambit. Mesh size in artery domains is 1 mm so that about 10,000 tetrahedral elements and 20,000 nodes are obtained. The nonuniform mesh is used to map the solid tissues. The mesh size 0.6 mm is used for the field close to active cryoprobe, where the large temperature gradient happens during cryosurgery. The mesh size 2 mm is used for the field far away from the active cryoprobe. Thus there are about 1,500,000 tetrahedral elements and 270,000 nodes for the whole solid tissues. After creating the mesh, Gambit generates an input file to the finite volume package Fluent 6.3, which has been extensively utilized to address a variety of practical engineering problems nowadays. The blood flow and temperature analysis are solved according to SIMPLE and second order up wind algorithms.

For every numerical simulation case, the steady blood velocity profile is firstly performed and considered as input parameters to solve the transient temperature field evolution. The second order upwind scheme is used to discretize the convectional term in Eq. (5). The first order implicit scheme is used for time evolution. The nonuniform time step is applied to improve the stability of numerical simulation. At the beginning of freezing, the temperature distribution near cryoprobe has large gradient and changes violently from 37 to − 196 °C in a short time. Thus the small time step is applied to improve the stability of numerical simulation.

6 $$\Delta t\, = \,\left\{ \begin{aligned} & 0.005\,s\quad \,\,\,t\, < \,1\,s \\ & 0.01\,s\quad \quad 1\,s\, \le \,t\, \le \,5\,s \\ & 0.05\,s\quad \quad 5\,s\, < \,t\, \le \,100\,s \\ & 0.1\,s\quad \quad \,\,\,100\,s\, < \,t \\ \end{aligned} \right.$$

## Results and discussion

### Steady blood velocity distribution

**Fig. 2:**
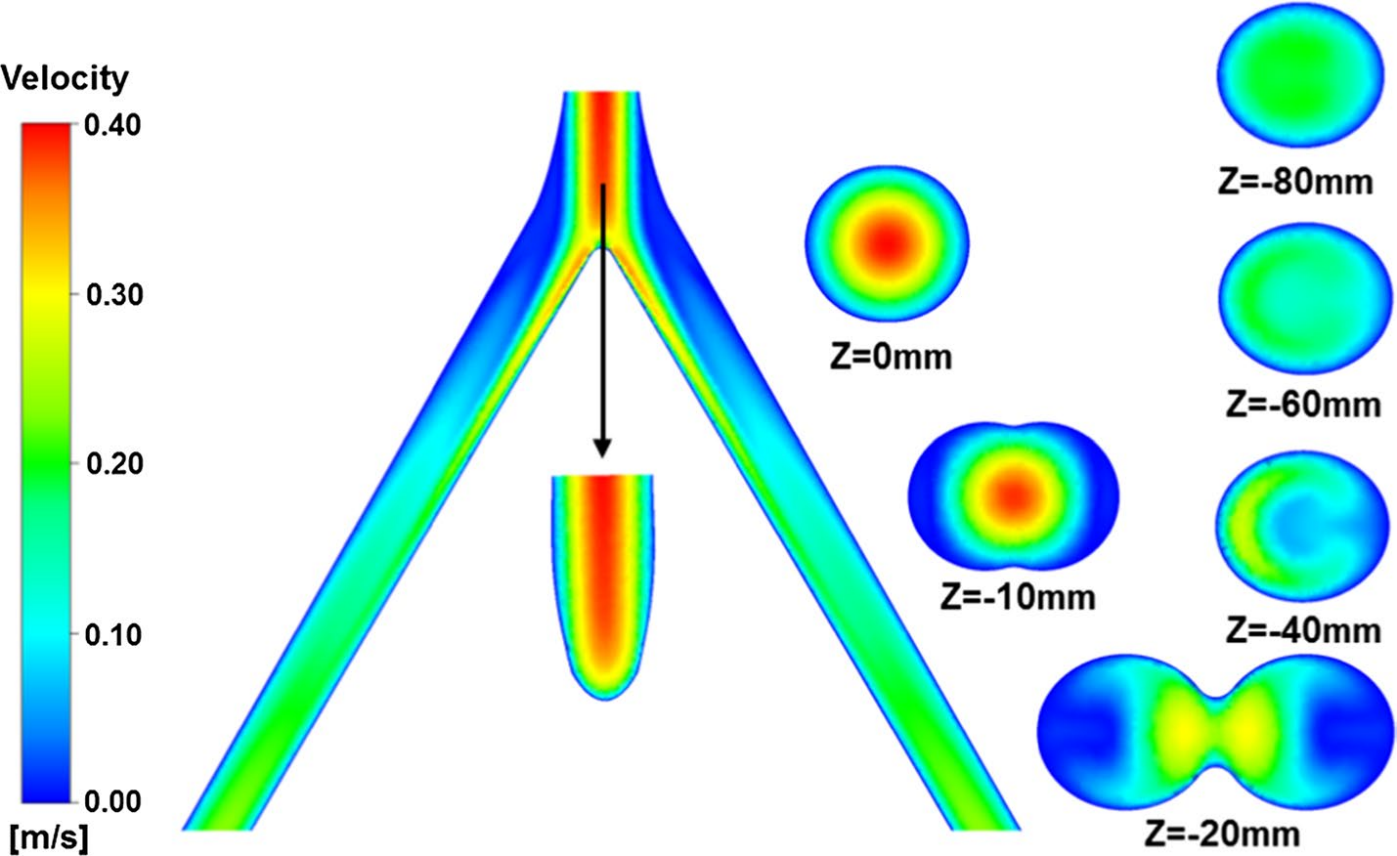
Contours of blood velocity magnitude in the planar plane: x = 0, y = 0 and different height cross-section planes of bifurcated artery, where *V0* = 0.20 m/s

Before solving the temperature evolution during cryosurgery, we have to obtain the velocity distribution in artery. Figure 2 shows the contours of blood velocity magnitude in x = 0, y = 0 and different height cross-section planes of bifurcated artery. A fully developed laminar blood with parabola velocity profile (V = 0.20 m/s) flows into the root vessel. With the increase of cross-section area, the amplitude of velocity parabola velocity distribution becomes weaker. When the blood flow approaches to bifurcation, the velocity is close to zero, which lead to a low velocity field near bifurcation. A boundary layer formed near the inside wall downstream from the flow bifurcation, with the maximum axial velocity just outside this domain. Another low velocity field happens at the outer wall of two daughter vessels inlet segment, where the blood flow presents the recirculation pattern. The blood flow entering into daughter vessels presents skewed structure toward the inner wall and develops finally a similar parabola profile. One can clearly find from Fig. 2 that compared to the simple blood flow with parabola pattern in single large line-like artery, the flow pattern in the bifurcation presents the complicated structure. This complexity would induce the inhomogeneous convective heat transfer between solid tissues and arterial bifurcation. The heat transfer would be enhanced in high velocity and weaken in low velocity fields.

### Thermal interaction between iceball evolution and arterial bifurcation

**Fig. 3:**
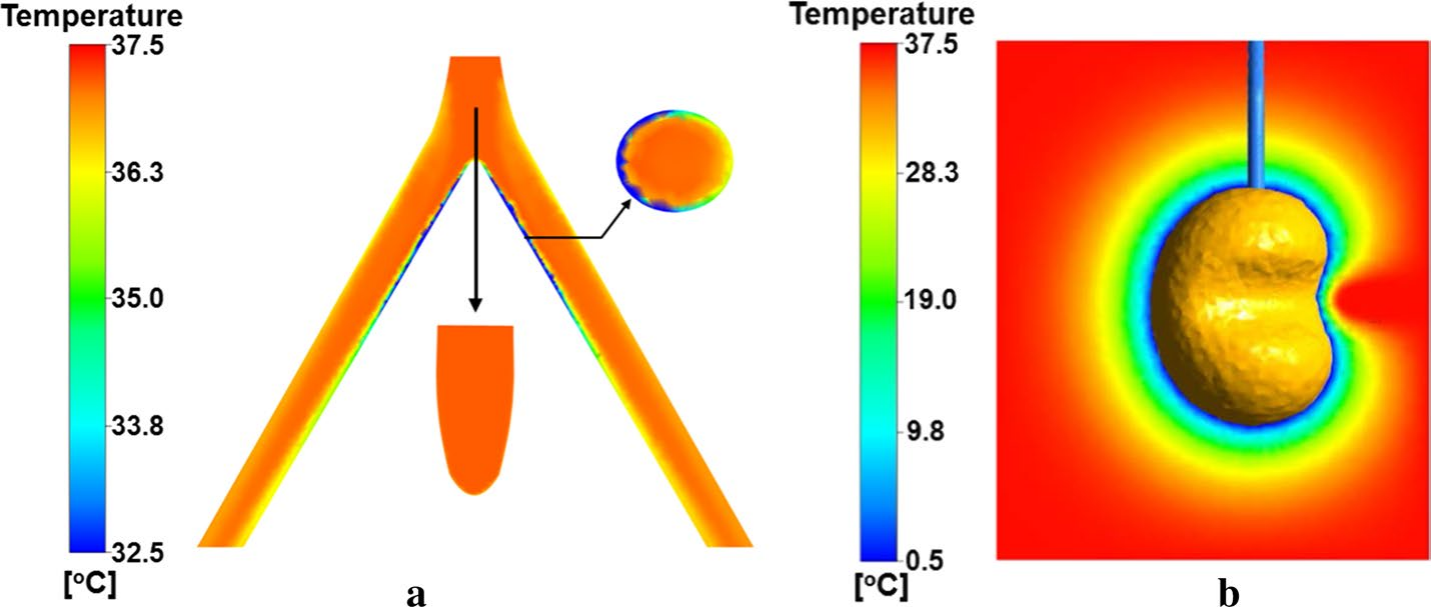
The temperature distribution in the plane x = 0, y = 0 and z = − 30 mm of bifurcated artery (**a**) and the iceball pattern view from x axis direction and temperature contours in the plane x = 0 (**b**) at freezing time t = 20 min (D_p_ = 4 mm) position for Ld = 20 mm

Firstly, a case with small distance L_d_ = 20 mm between cryoprobe and bifurcation point is considered here. Figure 3 shows the temperature distribution in the plane x = 0, y = 0 and z = − 30 mm of bifurcated artery (Fig. 3a) and the iceball pattern view from x axis direction and temperature contours in the plane x = 0 (Fig. 3b) at freezing time t = 20 min. The iceball boundary in present study is defined as iso-surface of T = 0 °C. In order to focus on the temperature variation in the whole bifurcated artery, the contour interval is here set as (32.5, 37.5 °C) (Fig. 3a). In fact, the lowest temperature for artery domains, which happens on the inner wall of branching artery closest to cryoprobe, could reach 5 °C. This temperature could not decrease with freezing time evolution. It indicates that the warm blood flow prevents the artery from being frozen. However, one should note that the blood in the artery may be frozen for a shorter distance L_d_ or a smaller inlet velocity of the root artery. The temperature in the vicinity of bifurcation point keeps at a relatively high level about 35 °C. From Fig. 3a, one can see that the bifurcated artery domains close to cryoprobe has a lower temperature distribution so that the following catchment area would be cooling. From Fig. 3b, we can see that the iceball stops evolution when encountering the arterial bifurcation. The large temperature gradient between iceball and artery wall is helpful to convective heat transfer which would enhance the thermal effects of blood flow. Different from line-like artery, the arterial bifurcation would lead to a more irregular iceball, which may make the target tissue frozen incompletely.

**Fig. 4:**
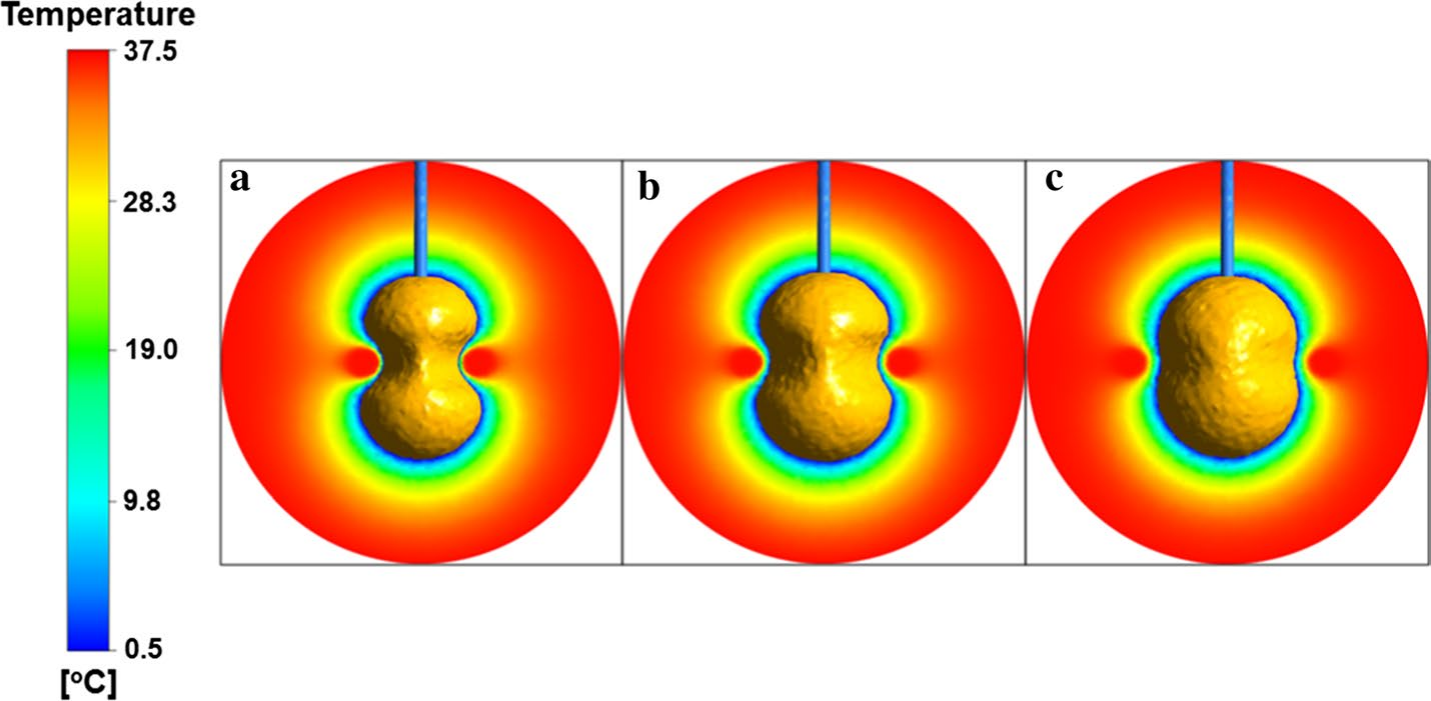
The iceball pattern view from z axis direction and temperature contours in the cryoprobe center plane for different cryoprobe (D_p_ = 4 mm) positions, **a** Ld = 20 mm, **b** Ld = 30 mm, **c** Ld = 40 mm, at freezing time t = 20 min

Figure 4 shows the iceball pattern view from z axis direction and temperature contours in the center plane of cryoprobe (D_p_ = 4 mm) for its different positions, L_d_ = 20 mm (Fig. 4a), L_d_ = 30 mm (Fig. 4b) and Ld = 40 mm (Fig. 4c) at freezing time t = 20 min. It is easy to find from Fig. 4 that the intensity of thermal interaction between iceball and artery bifurcation becomes weaker with increase of the relative distance between cryoprobe and bifurcation. The shorter distance between cryoprobe and artery would advance their thermal interaction during freezing. The iceball formation induced by cryoprobe has a limited volume. Thus the thermal effects from warm blood flow on the iceball would disappear when their distance is beyond available range. Clinical practice for cryosurgery has demonstrated that the most serious cryoinjury is achieved when the target tissue undergo below so-called lethal temperature, which is considered currently as in the range of − 50 to − 40 °C [3]. Table 1 presents the volume of iceball and lethal domains (where the temperature is below − 40 °C) for different cryoprobe positions. Both the volume of iceball and lethal domain increase when the cryoprobe is far away from arterial bifurcation.

**Table 1:** The iceball volume (T = 0 °C) and the lethal volume (T = − 40 °C) for different cryoprobe positions at freezing time t = 20 min

Cryoprobe position (mm)	Ld = 20	Ld = 30	Ld = 40
Iceball volume (cm^3^)	39.13	41.90	51.90
Lethal volume (cm^3^)	6.81	8.33	9.48

**Fig. 5:**
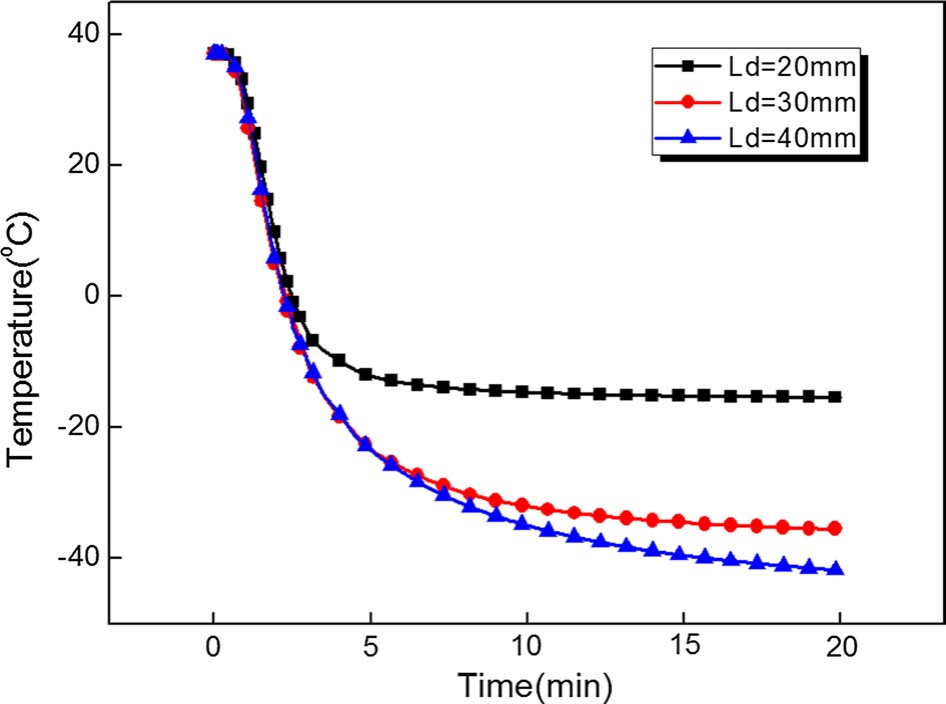
The temperature evolution of the point (located on the line Ld) with distance 12 mm from cryoprobe centerline for different cryoprobe (D_p_ = 4 mm) positions Ld = 20 mm, Ld = 30 mm and Ld = 40 mm

**Fig. 6:**
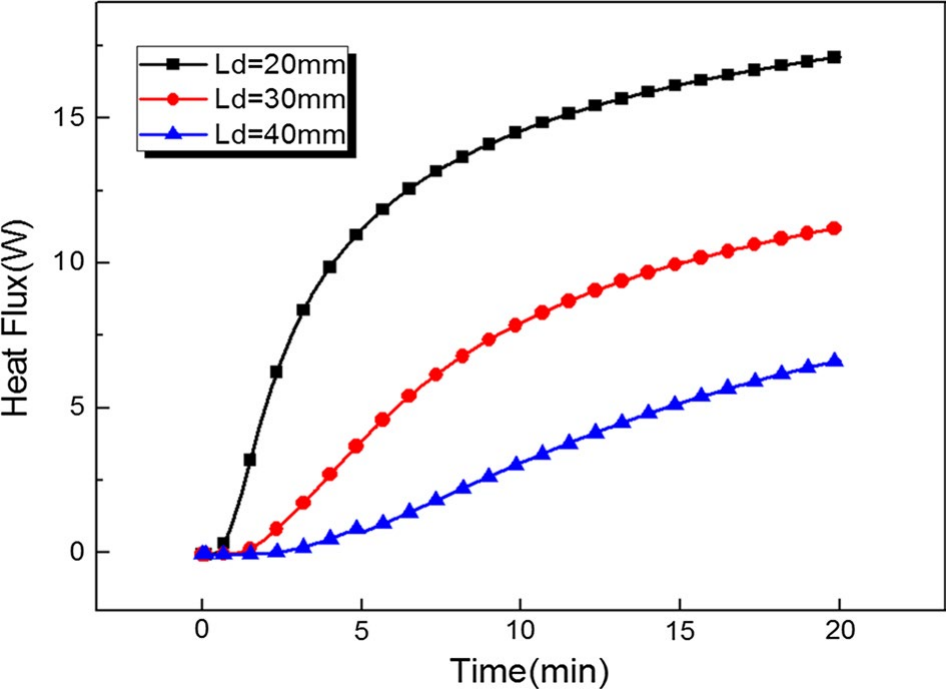
The total heat flux evolution of bifurcated artery surface for different cryoprobe (D_p_ = 4 mm) positions Ld = 20 mm, Ld = 30 mm and Ld = 40 mm

Figure 5 records the temperature evolution of the point (located on the center-line of cylinder) with a distance 12 mm from cryoprobe centerline for different cryoprobe (D_p_ = 4 mm) positions L_d_ = 20 mm, L_d_ = 30 mm and L_d_ = 40 mm. The temperature remains at a high level for L_d_ = 20 mm due to strong thermal effects of warm blood flow. At the beginning of the freezing process, the temperature of the recorded point has the consistent cooling rate for each position. Such consistence would keep longer time with increase of L_d_. The reason is that at freezing initial stage a large distance between the iceball and artery leads to a weak thermal interaction. Thus the temperature of the recorded point close to cryoprobe is mainly determined by iceball at initial freezing stage. Figure 6 shows the total heat flux evolution of bifurcated artery surface for different cryoprobe (D_p_ = 4 mm) positions. With increase of Ld, the total heat flux of bifurcated artery surface significantly decrease. This is mainly because the longer distance weaken the thermal effects of the blood flow.

**Fig. 7:**
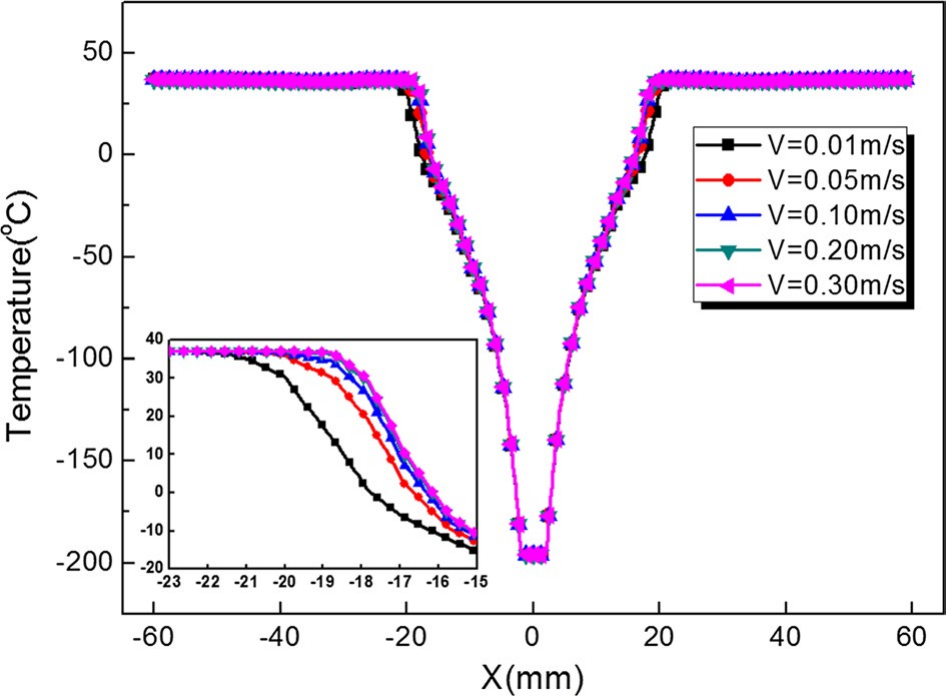
The temperature distribution of the line: ([− 60 60], 0, − 50) mm for different inlet velocity V = 0.01, 0.05, 0.10, 0.20 and 0.30 m/s with cryoprobe position Ld = 30 mm and freezing time t = 20 min

**Fig. 8:**
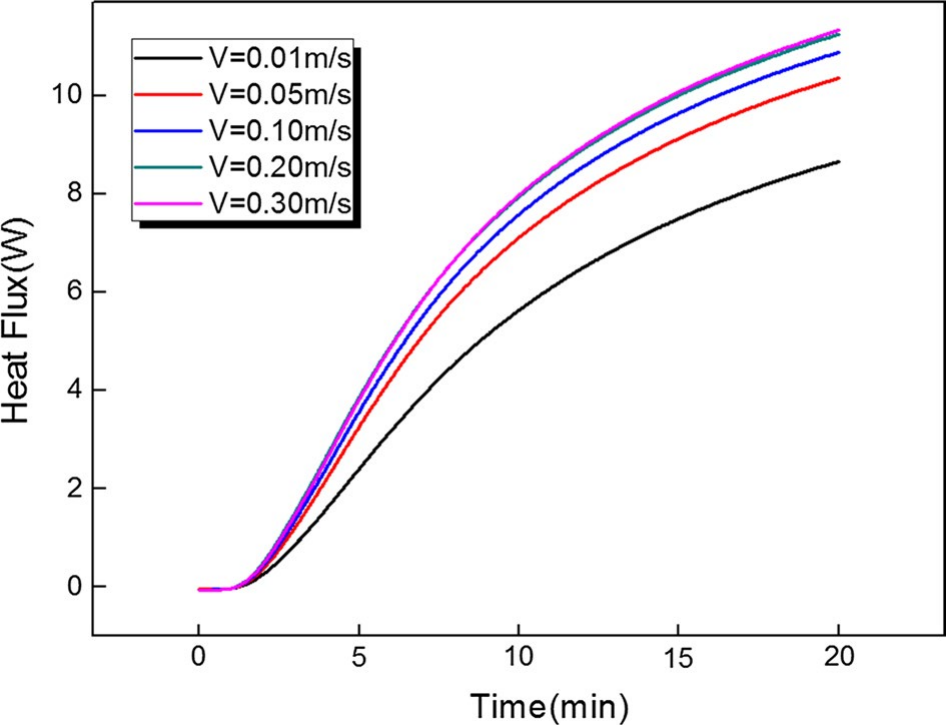
The total heat flux evolution of bifurcated artery surface for different inlet velocity V = 0.01, 0.05, 0.10, 0.20 and 0.30 m/s with cryoprobe position Ld = 30 mm

In order to evaluate the effects of arterial bifurcation on the iceball growth, different inlet velocities of artery root are considered. Figure 7 represents the temperature distribution of the line: ([− 60 60], 0, − 50) mm for different inlet average velocities V = 0.01, 0.05, 0.10, 0.20 and 0.30 m/s with the same cryoprobe position Ld = 30 mm and freezing time t = 20 min. Figure 8 illustrates the total heat flux evolution of bifurcated artery surface for different inlet average velocities. Both Figs. 7 and 8 indicate that the smaller velocity would induces more weak thermal effects of arterial bifurcation. In addition, with inlet velocity increase, the artery thermal effects become flats, such as cases V = 0.20 and 0.30 m/s. The results are also demonstrated by evaluating both the iceball and lethal volumes for different inlet velocity cases (shown in Table 2).

**Table 2:** The iceball volume (T = 0 °C) and the lethal volume (T = − 40 °C) for different inlet velocity with the same cryoprobe position L_d_ = 30 mm and freezing time t = 20 min

Inlet velocity (m/s)	V = 0.01	V = 0.05	V = 0.10	V = 0.20	V = 0.30
Iceball volume (cm^3^)	44.32	42.59	42.00	41.90	41.83
Lethal volume (cm^3^)	8.56	8.41	8.35	8.33	8.32

For cryosurgery practice, except for killing tumor cells, another important task is to prevent organs and large vessels from being damaged. Table 3 lists the treatment time of cryosurgery when the minimum temperature of the bifurcated artery surface approaches to the freezing temperature (T = 0 °C) with different cryoprobe positions and inlet velocities of artery root. As can be seen from Table 3, different cryoprobe positions and inlet velocities of artery root have strong impacts on the treatment time of cryosurgery. When Ld equals to 10 mm and 15 mm, the treatment time of cryosurgery should not exceed 65 s. And when the large cryoprobe distance is adopted, the bifurcated artery could avoid the injury owing to the thermal effects of blood flow.

**Table 3:** The treatment time of cryosurgery when the minimum temperature of the bifurcated artery surface approaches to the freezing temperature (T = 0 °C) according to different cryoprobe positions and inlet velocities of artery root

	Ld = 10 (mm)	Ld = 15 (mm)	Ld = 20 (mm)	Ld = 30 (mm)	Ld = 40 (mm)
V = 0.01 (m/s)	9.93 s	40.50 s	109.15 s	731.50 s	∞
V = 0.05 (m/s)	11.10 s	51.80 s	296.40 s	∞	∞
V = 0.10 (m/s)	11.40 s	58.05 s	∞	∞	∞
V = 0.20 (m/s)	11.50 s	61.90 s	∞	∞	∞
V = 0.30 (m/s)	11.60 s	65.30 s	∞	∞	∞

∞ means the treatment time more than 20 min

**Fig 9:**
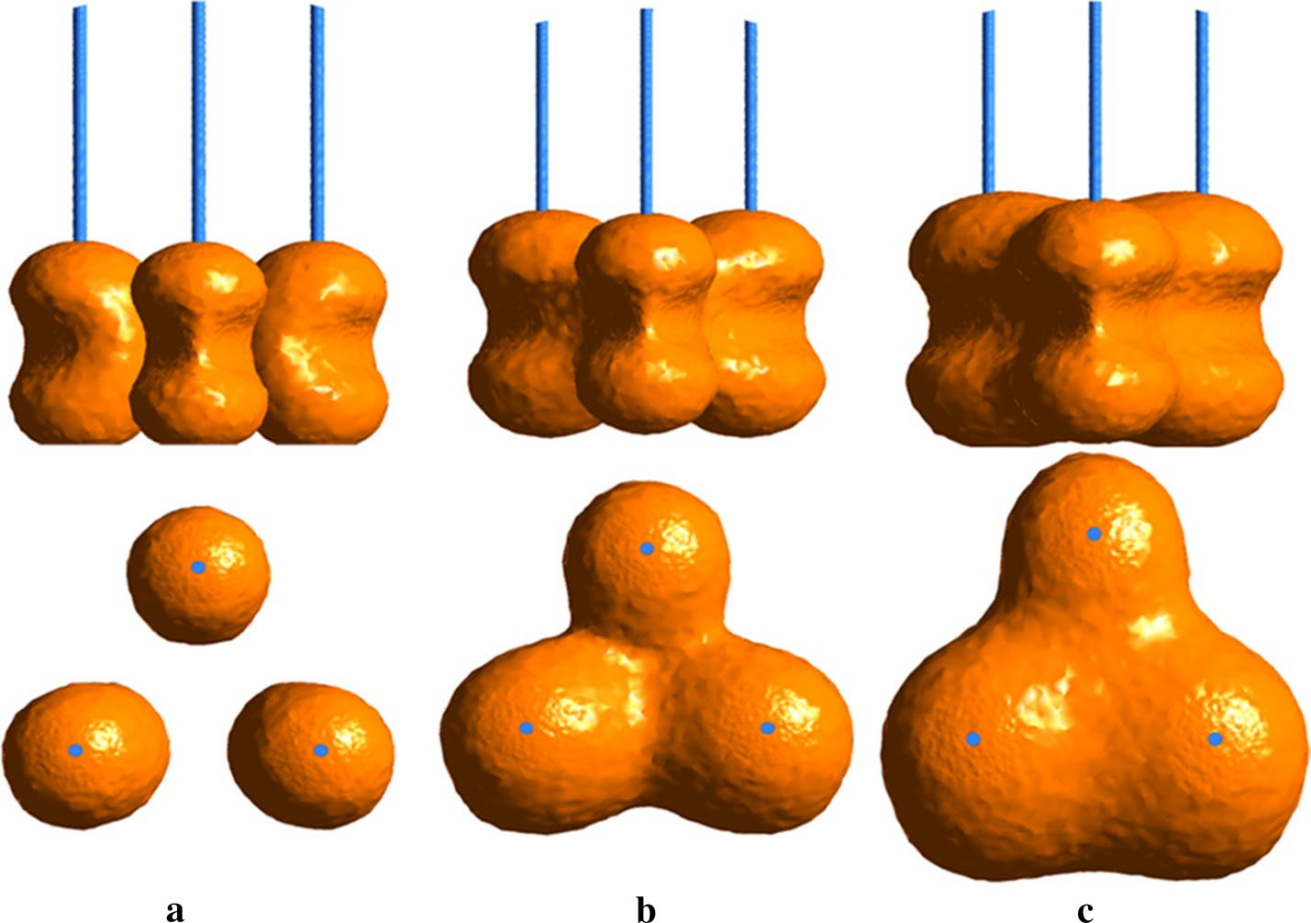
The iceball view form x direction and y direction for three cryoprobes with freezing evolution: **a** t = 5 min, **b** t = 15 min, **c** t = 25 min, other parameters D_p_ = 2 mm, where V = 0.20 m/s

Single cryoprobe often fails to overcome the large and irregular diseased tissues. Here, three cryoprobes array is used to freeze large fields. Three cryoprobes with diameter D_p_ = 2 mm and active length L_p_ = 20 mm are distributed uniformly on the circumference with radius 20 mm. Its center locates on the center-line of cylinder with L_d_ = 30 mm. Figure 9 shows that the iceball view from x direction and y direction for three cryoprobes at different freezing time. Three single iceballs are formed at initial cooling stage and combined fast to a large ingle iceball under a high-strength freezing. It can be seen from Fig. 9 that the three iceball induced by different cryoprobes have different growth rates due to varied cryoprobe positions and the inhomogeneous convective heat transfer between solid tissue and arterial bifurcation.

**Fig 10:**
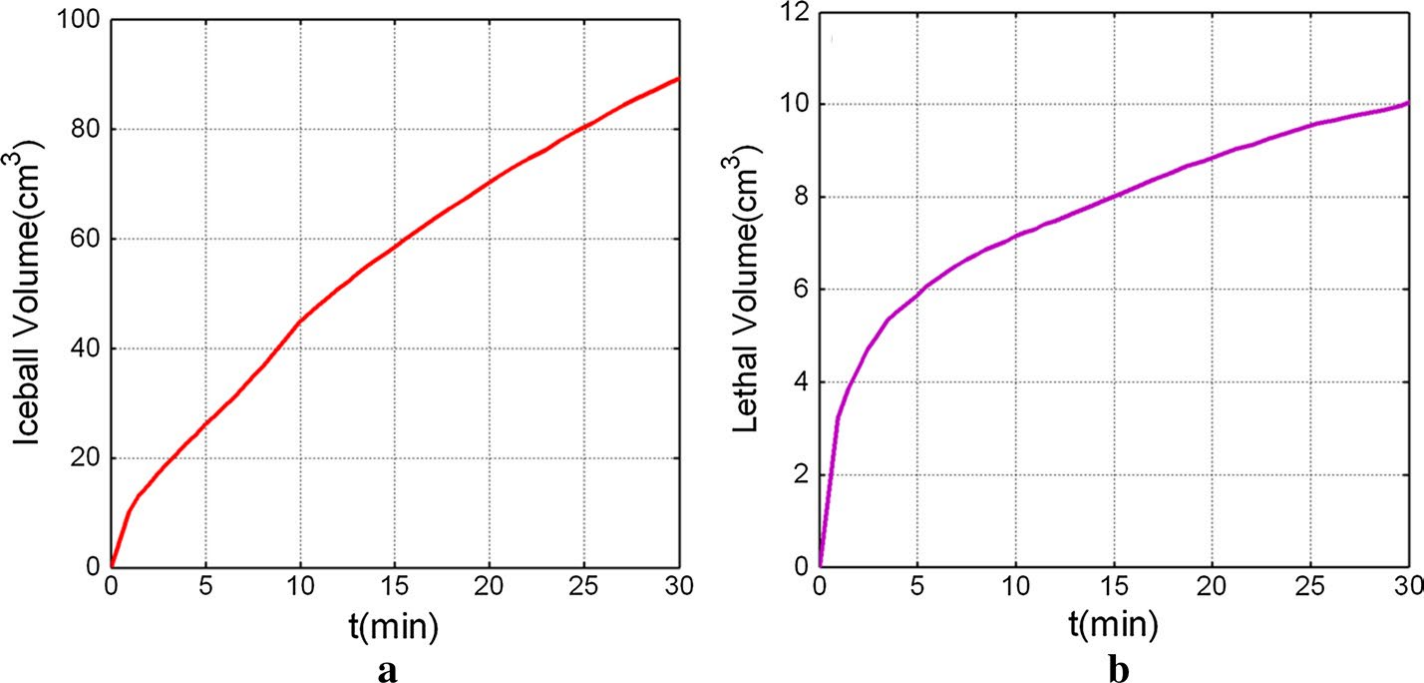
The iceball volume **a** and lethal volume **b** for three-cryoprobe cryosurgery for freezing time evolution

Figure 10 shows the iceball volume and lethal volume for three cryoprobes cryosurgery with freezing time evolution. Both iceball and lethal volumes’ growth rate is high at initial freezing stage and becomes lower at later freezing stage. In fact, this growth rate would approach zero after a longer freezing time, while it could not be observed in present short freezing time.

## Conclusions

In summary, the present paper has adopted three dimensional numerical simulation method to investigate the thermal effects of arterial bifurcation on temperature responses during cryosurgery based on single and multiple cryoprobe system. We have investigated in detail that the blood velocity distribution in arterial bifurcation and its effects on the iceball growth. The results indicate that complex blood velocity distribution could induce the inhomogeneous convective heat transfer between solid tissue and arterial bifurcation. Thus the iceball near arterial bifurcation presents strong irregular geometry. The blood flow of bifurcated artery has significant heating effects on the target freezing domains. It is also noteworthy that the artery wall is easily suffering from cold injury, which should be paid by special attention. In order to protect the artery wall, nanoparticle and external fields [19, 20] could be applied to enhance heat transfer near artery wall with low temperature.
